# Glutathione Ethyl Ester Supplementation during Pancreatic Islet Isolation Improves Viability and Transplant Outcomes in a Murine Marginal Islet Mass Model

**DOI:** 10.1371/journal.pone.0055288

**Published:** 2013-02-12

**Authors:** Alexandre S. Raposo do Amaral, Rena L. Pawlick, Erika Rodrigues, Flavia Costal, Andrew Pepper, Flávio H. Ferreira Galvão, Maria Lucia Correa-Giannella, A. M.James Shapiro

**Affiliations:** 1 Alberta Diabetes Institute, University of Alberta, Edmonton AB, Canada; 2 Laboratório de Endocrinologia Celular e Molecular (LIM-25) do Hospital das Clínicas da Faculdade de Medicina da Universidade de São Paulo, São Paulo, Brazil; 3 Unidade de Transplante e Cirurgia de Fígado (LIM37), Departamento de Gastroenterologia da Faculdade de Medicina da Universidade de São Paulo, São Paulo, Brazil; Universidad Miguel Hernández de Elche, Spain

## Abstract

**Background:**

The success of pancreatic islet transplantation still faces many challenges, mainly related to cell damage during islet isolation and early post-transplant. The increased generation of reactive oxygen species (ROS) during islet isolation and the consumption of antioxidant defenses appear to be an important pathway related to islet damage.

**Methodology/Principal Findings:**

In the present study we evaluated whether supplementation of glutathione-ethyl-ester (GEE) during islet isolation could improve islet viability and transplant outcomes in a murine marginal islet mass model. We also cultured human islets for 24 hours in standard CMRL media with or without GEE supplementation. Supplementation of GEE decreased the content of ROS in isolated islets, leading to a decrease in apoptosis and maintenance of islet viability. A higher percentage of mice transplanted with a marginal mass of GEE treated islets became euglycemic after transplant. The supplementation of 20 mM GEE in cultured human islets significantly reduced the apoptosis rate in comparison to untreated islets.

**Conclusions/Significance:**

GEE supplementation was able to decrease the apoptosis rate and intracellular content of ROS in isolated islets and might be considered a potential intervention to improve islet viability during the isolation process and maintenance in culture before islet transplantation.

## Introduction

The major changes introduced in the last decade have optimized the clinical outcomes and highlighted the role of pancreatic islet transplantation in the treatment of type 1 diabetes [1]. However, the success of pancreatic islet transplantation still faces many challenges, mainly related to cell damage during islet isolation and early post-transplant [2]–[4]. Several factors during the isolation may contribute to the decrease of islet viability including ischemia, changes in temperature, exposure to collagenase and pancreatic enzymes, mechanical disruption, and osmotic stress [5], [6]. The increase in reactive oxygen species (ROS) generation and the consumption of antioxidant defenses might be a common factor related to these injuries.

Glutathione (GSH) is a tri-peptide synthesized from glutamate, cysteine and glycine found in almost all human cells. It has a central relevance in physiological antioxidant defenses reacting with ROS and forming oxidized GSH, which can be reduced again or removed from the cell in the oxidized form by a membrane transporter [7], [8].

GSH is considered an essential compound of University of Wisconsin (UW) solution [9], [10] and plays a major role on the protective effects of this preservation solution [11]. Several studies have shown the benefits of GSH during ischemic conditions on liver [12], heart [13], [14], and brain [15]. Precursors of GSH, like N-acetylcysteine, also might have a positive impact [16].

ROS produced under ischemic conditions may contribute to functional impairment of isolated islets and graft loss at the early stages of islet transplantation [17]. Tonooka et al. showed very low concentrations of GSH peroxidase after human islet isolation [18], and glutamate, a precursor of GSH, showed positive effects when used during digestion on human islet isolation [19].

GSH has been used in past studies of organ preservation, but surprisingly the ethyl-ester form of this molecule (GEE) has much more potential benefit. A minor change to the solubility of GSH, can have a major impact on how effective it is. GSH is not taken up by cells in its intact form while its esterification by an ethyl-ester increases GSH bioavailability by rendering it a lipid soluble molecule able to cross cell membrane [20]. Thus, the aim of the present study was to evaluate whether supplementation with the glutathione-ethyl-ester (GEE) could improve islet viability and efficacy in a murine marginal islet transplantation model. We also evaluated the effect of GEE in the apoptosis rate of cultured human islets.

## Materials and Methods

### Animal Care and Use

BALB/c mice were obtained from Charles River Laboratory and housed under conventional conditions. All animals were cared for according to the guidelines of the Canadian Council on Animal Care, and ethical approval was obtained from the animal welfare committee at the University of Alberta.

### Mouse Islet Isolation

Mouse islets were isolated using established protocols [21]. All reagents were obtained from Sigma Aldrich unless otherwise specified. In brief, mice were sedated with avertine and prepared for surgery. A total of 10 mice were allocated in the control and treated groups for each isolation. A 27 gauge needle was positioned inside the common bile duct and the pancreas was distended using collagenase Type V solution (1.0 mg/ml in Hanks’ buffered saline solution [HBSS]) in the control group or collagenase solution + *glutathione ethyl-ester (GEE)* (1.0 mg/ml in HBSS + 10 mM GEE) in the treated group. Once the pancreas was distended, the animal was euthanized. The distended pancreas was placed in a 50 ml conical tube filled with 15 mL cold HBSS (10 pancreas/tube). The distensions were made alternately between the groups and the whole process was completed within 1 hour. Once all pancreases were distended, they were transferred to cold collagenase solution in the control group or cold collagenase solution +10 mM GEE in the treated group. The tubes were placed in a shaking water bath for 15 minutes at 150 rpm (Julabo model SW22). After digestion, islets were purified with Histopaque-density centrifugation. Handpicked islets were washed with HBSS, counted, then placed in short-term culture (1 hour) in CMRL-1066 (Mediatech, Manassas, VA) supplemented with 10% fetal bovine serum (FBS), L-glutamine (100 mg/l), penicillin (112 kU/l), streptomycin (112 mg/l) and HEPES (25 mmol/l).

### Glutathione Ethyl-ester (GEE) Supplementation

Glutathione monoethyl-ester (Sigma Aldrich) was weighed and added to collagenase solution before pancreas distension at a concentration of 10 mM. The control group pancreases were distended and digested in the standard collagenase solution and the treated group pancreases were distended and digested in the 10 mM GEE supplemented collagenase solution. The was no supplementation of GEE during the other stages of the isolation process.

### Measurement of Reactive Oxygen Species

Intracellular ROS was measured by oxidation-sensitive fluorescent probe Carboxy-H_2_DCFDA (Molecular Probes). The dye has excitation/emission of approximately 495/529 nm. After isolation, islets were dissociated using Accutase (Innovative Cell Technologies, Inc, San Diego, CA) at 37°C. FBS was added to stop the process after 10 min. Single cell suspensions were incubated in the dark for 30 min at 37°C. After washing, the cells were analyzed by flow cytometry using a BD LSR II (BD Biosciences). Data were analyzed using the FCS Express 3 software (DeNovo Software).

### Membrane Integrity

Membrane integrity was assessed using SYTO Green 13 Fluorescent Nucleic Acid Stain (Molecular Probes, Cedarlane Labs, Hornby ON, Canada) and ethidium bromide (Sigma-Aldrich, Oakville ON, Canada). A total of 100 islets was viewed under a Nikon Eclipse TE300 fluorescent microscope fitted with a 50-Rhodamine filter (Mississauga ON, Canada).

### Fractional Beta-cell Viability

After isolation, islets were dissociated using Accutase (Innovative Cell Technologies, Inc, San Diego, CA) at 37°C. FBS was added to stop the process after 10 min. Single cell suspensions were incubated with 3 µM Newport Green (NG, Molecular Probes) and 0.2 nM of Tetramethylrhodamine ethyl ester (TMRE, Molecular Probes) for 45 min at 37°C in PBS. After washing, 1 mg/ml of 7-AAD(Molecular Probes) was added [22]. Cell suspension was analyzed by flow cytometry using a BD Laser Scanning Cytometer II (LSR II, BD Biosciences). Data were analyzed using the FCS Express 3 software (DeNovo Software).

### Dead End Fluorimetric TUNEL

Apoptosis was quantified on isolated islets, 24 h grafts and 30 days grafts using TUNEL staining (DeadEnd Apoptosis Detection System, Promega, Madison, WI) Nuclear counter staining with DAPI (Molecular Probes, Eugene, OR) was used to detect all cells present in the sample. Islets were preserved on formalin fixative and embedded in paraffin. Islet grafts were harvested, placed in formalin, processed and embedded in paraffin. To quantify of apoptosis *in vivo* a 200× magnification was used. The number of TUNEL positive cells (green) within the insulin positive islet graft area (red) of the section was counted and compared to the total number of DAPI positive nuclei (blue) within that same field to determine percent apoptosis. Sections were prepared from recipients in each cohort and at least 3 fields were analyzed in each section.

### Glucose-stimulated Insulin Release

Triplicate aliquots containing 50 mouse islets were left in culture for 24 h in CMRL media (supplemented with 5.6 mM of D-glucose and 10% FBS). Islets were washed 3 times in RPMI medium supplemented with 10% BSA and allowed to gravity settle for 5 min. The media was replaced with low-glucose containing RMPI medium (2.8 mM) and incubated for 1 h at 37°C, followed by incubation in high-glucose containing RPMI medium (20 mM). The supernatant was collected and stored at −20°C. Insulin was quantified using an ELISA kit (Alpco Diagnostics, Windham, NH). In each experimental condition, the fold stimulation was calculated by dividing the mean insulin released from islets cultured in the high-glucose medium by the mean insulin released from islets cultured in the low-glucose medium in parallel.

### Islet Transplantation

Streptozotocin was administered to recipient mice to induce diabetes (200 mg/kg intra peritoneal). Animals were considered diabetic after 2 consecutive blood glucose measurements ≥20 mmol/l using a OneTouch Ultra glucometer (Lifescan). The transplants were performed immediately after islet isolation. For mouse islet studies, a non marginal mass of 500 islets and a marginal mass of 150 islets were implanted into the kidney sub capsular space. For 24 h studies, 300 islets were transplanted under the kidney capsule. Normoglycemia were considered as two measurements of blood glucose <11 mmol/l.

### Glucose Tolerance Test

Transplanted mice were fasted for 16–18 h and injected intraperitoneally with 25% dextrose at 3 mg/kg body weight. Blood glucose levels were analyzed at baseline, 15, 30, 60, 90 and 120 min post injection.

### Graft Insulin Content

Islet grafts were harvested from the kidney capsule and stored at –80°C until bulk analysis could be performed. Extraction was performed in acid-ethanol by homogenization and ultrasonic cell membrane disruption. Insulin concentration of the neutralized extract was measured using a commercial ELISA kit (Alpco Diagnostics, Windham, NH).

### Cultured Human Islets

Human cadaveric pancreases were removed from brain dead multi-organ donors following *in situ* vascular flushing with cold UW solution and transported to the clinical islet isolation laboratory. Upon arrival at the laboratory, the pancreatic duct was cannulated and liberase enzyme (Roche Diagnostics, Indianapolis, IN) perfused. The pancreas was enzymatically and mechanically dissociated before the islets were separated on a refrigerated Cobe 2991 centrifuge (Cobe BCT, Lakewood, CO) and placed in culture [1], [23]. All protocols were approved by the health research ethics board of the University of Alberta. All human tissue samples were de-identified and analyzed anonymously prior to analysis. Written informed consent was given for their use in research. Human islets were made available for these studies after failure to achieve sufficient islet yield for clinical transplantation [24], but after a period of culture in standard insulin-transferrin-selenium supplemented CMRL 1066. Therefore for these studies, GEE exposure was only possible during a period of *in vitro* culture, and it was not possible to add GEE to the collagenase perfusion system during the digestion phase of the isolation, in contrast to the mouse studies.

Samples of five different human pancreases were obtained after islet isolation and remained in culture from 10 to 50 h before being or not exposed to different GEE concentrations.

Sample of islets from three different pancreases were aliquoted and cultured for 24 h in standard CMRL media with or without supplementation with 10 mM GEE. Samples of other two pancreases were submitted to five different concentrations of GEE during 24 h culture (0, 5, 10, 15 and 20 mM). Islets were then evaluated regarding ROS content, membrane integrity, fractional beta-cell viability and TUNEL, as described above.

### Statistics

Data were analyzed using GraphPad Prism (Version 3.0, San Diego, CA). *P* values less than 0.05 were considered statistically significant. Graphical representation of data is represented as mean ± SEM, unless otherwise indicated in the figure legend. Wilcoxon signed rank test was used for paired analysis and Mann-Whitney test for unpaired analysis. Survival analysis was carried out using log-rank analysis. The ANOVA test with Tukey post test were used for the experiment with different GEE concentrations on cultured human islets.

## Results

### Mouse Islets

Distension of the pancreases was alternated between control and treated groups. The difference between the groups was the supplementation of GEE during distension and digestion on treated pancreases. A total of 14 isolations were performed and there was no significant difference in the number of isolated islets between the groups (Control 1,661±124 islets/10 mice vs. GEE 1,774±156 islets/10 mice, p  = 0.1230). Immediately after isolation, islets were aliquoted for *in vitro* and *in vivo* analysis.

**Figure 1:**
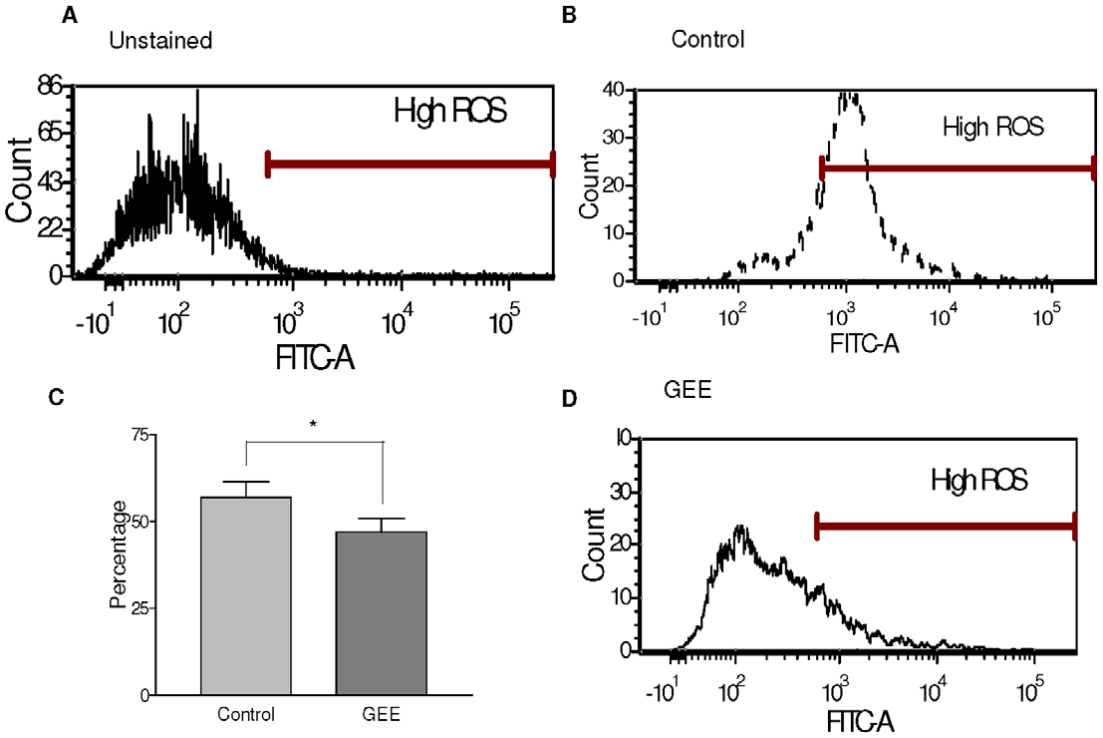
Intracellular reactive oxygen species after islet isolation. There is a greater percentage of increased fluorescence intensity (High ROS) measured by the excitation and emission of carboxy-h2DCFDA at 495/529 nm (FITC channel), in the control mouse islets (B) when compared to the GEE treated islets (D). ROS content in the control cells (56.9+/−4.33) is significantly higher than in the treated cells (46.98+/−3.94) (p<0.005) (C). Panel A is depicting unstained islets. N = 7 islet isolations.

We thus tested whether ROS contents were altered by GEE treatment, by measuring oxidation of the redox-sensitive dye diclhorodihydrofluorscein diacetate (carboxy-H2DCFDA). There was a decrease in the intracellular ROS content on pancreatic islets isolated with GEE. (Fig. 1. Control 57±4.3% vs GEE 47±3.9%; p<0.05).

**Figure 2:**
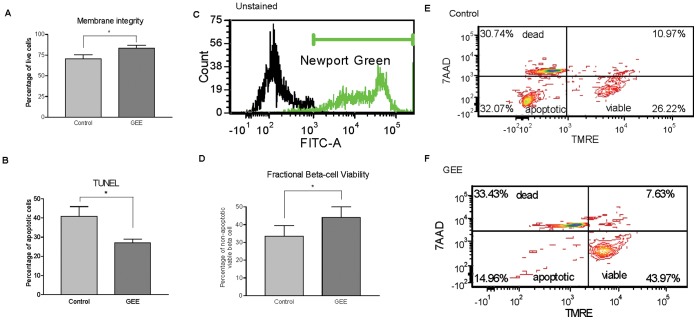
Islet viability *in vitro*. A decrease in islet damage was observed in different assays after islet isolation supplemented with 10 mM GEE. (A) Membrane integrity evaluated with Syto Green/Ethidium Bromide (P<0.05). (B) Islet apoptosis was measured with TUNEL staining after islet isolation. (P<0.05). (C, D, E and F) Fractional beta-cell viability staining using Newport Green, 7-aminoactinomycin D (7-AAD) and tetramethylrhodamine ethyl ester (TMRE) (P<0.05).

Necrosis is characterized by loss of membrane integrity, cell lysis and the generation of a local inflammatory reaction. The cell membrane integrity and subsequent cell viability were measured by Syto Green and Ethidium Bromide staining. There were a higher number of viable islets (green) in the treated group *versus* the control group (Fig. 2, Control 70.6±3.4% vs GEE 83.6±4.8%, p<0.05).

Histological analysis of apoptosis was performed using DeadEnd Fluorometric TUNEL staining. DNA fragmentation is a landmark of apoptosis and was detected on fixed islets by co-staining tissue insulin with the TUNEL system. A larger number of TUNEL-positive islet cells was observed in control islets than in treated islets (Fig. 2, Control 39.2±5.0% *vs* GEE 29.0±1.9%, p<0.05).

Beta cell content and fractional viability were measured by flow cytometry analysis using the fluorescent dyes: Newport Green, TMRE and 7-aminoactinomycin D (7-AAD) [22]. The Newport Green dye allowed for the selection of beta cells based on their zinc content. TMRE binds to the mitochondrial membrane showing low staining on apoptotic cells and 7-AAD measures membrane permeability in dead cells. A higher number of viable beta-cells (Newport Green positive/TMRE positive/7-AAD negative) was observed in the treated group (Fig. 2, Control 21.4±3.4% *vs* GEE 33.7±3.9%, p<0.05).

Triplicate aliquots of islets from each group were placed on culture for 24 h after isolation, then, a glucose-stimulated insulin release assay was performed. We were not able to find a statistically significant difference between the groups based on their stimulation index (Control 3.2±0.5 *vs* GEE 4.1±0.8, P = 0.57).

To test the efficacy of isolated islets *in vivo*, we transplanted islets on the renal capsule of diabetic mice. A non marginal mass of 500 islets and a marginal mass of 150 islets were transplanted. Seven transplants were performed on non marginal group, 20 transplants on control marginal mass group and 23 transplants on GEE treated marginal mass group. A large group was needed in the minimal mass model to achieve a sufficient number of cured animals to perform IPGTT and to the graft histologically and for graft insulin content analysis.

At the end of follow-up, the mean blood glucose was 7.23±0.30 mmol/L in the group transplanted with 500 islets and 6.48±0,52 mmol/L in the group transplanted with 500 GEE islets. In the marginal mass group (500 islets), the mean blood glucose level was 18.54±1.48 mmol/L, in the non-marginal control group (150 islets) and 12.43±1.54 mmol/L in the non-marginal GEE group (150 islets) (p<0.05).When transplanted with 500 islets, all the animals reached normoglycemia and there was no significant difference on the evolution of blood glucose between the control and treated islets.

**Figure 3:**
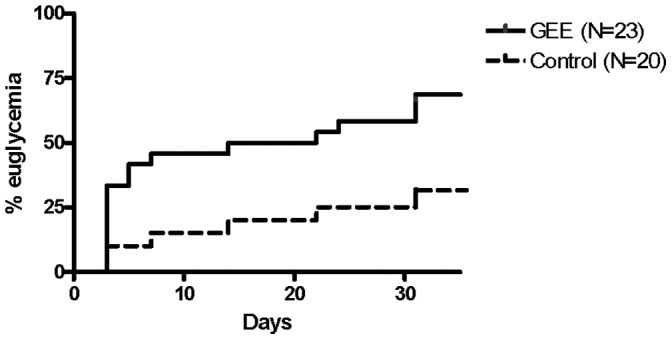
Percentage of normoglycemic mice after marginal mass transplant (150 islets transplanted under the renal cavity in diabetic, streptozotocin treated mice) with islets treated (GEE) or not (Control) with glutathione-ethyl-ester (P<0.05).

However after transplantation with a marginal mass of islets, a percentage significantly higher of animals reached normoglycemia in the group which received islets treated with GEE in comparison to the group which received untreated islets (Fig. 3, Control 30% vs GEE 65.2%, P<0.05).

**Figure 4:**
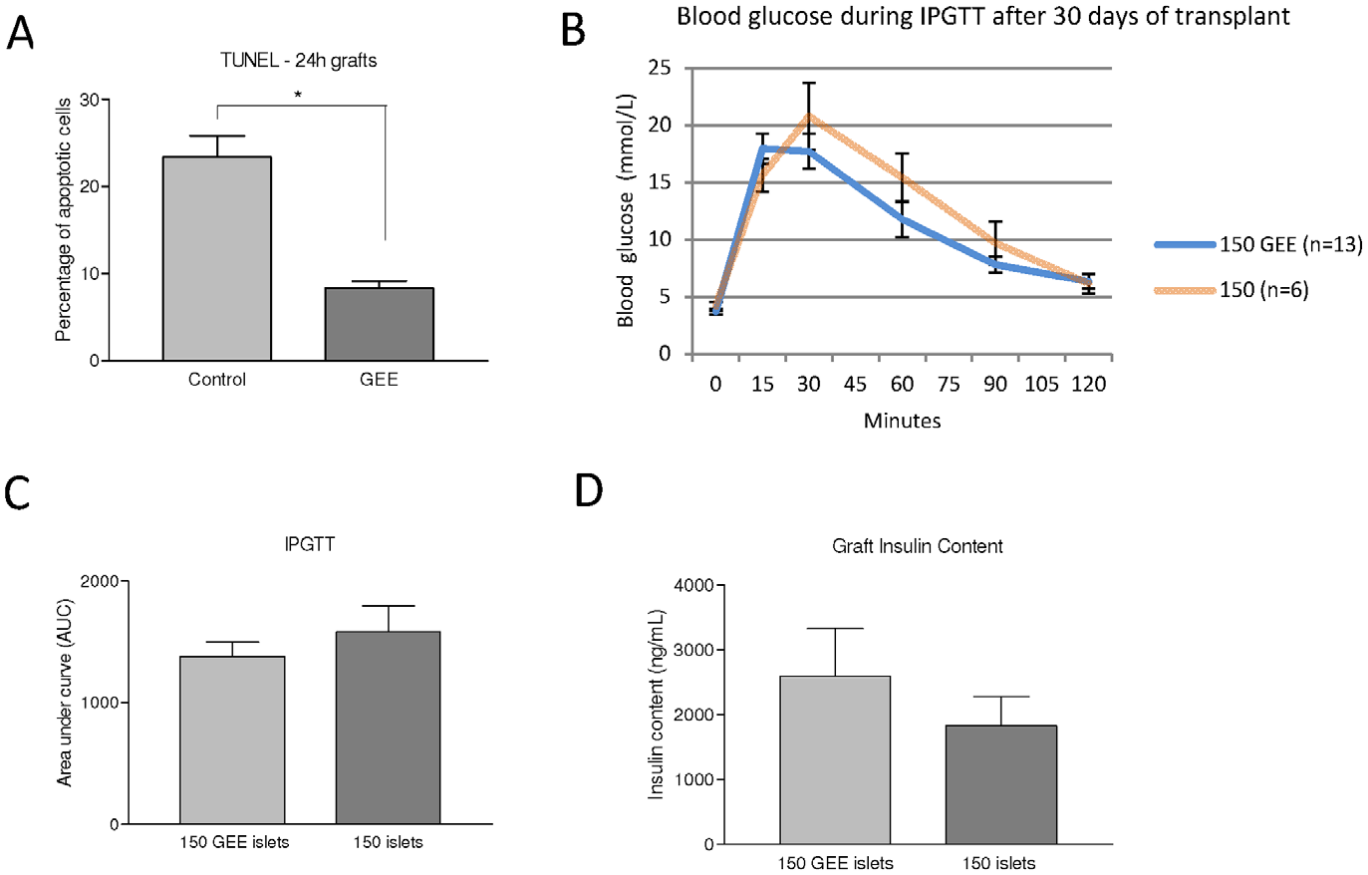
Islet viability *in vivo*. (A) Percentage of TUNEL positive cells from 24 h grafts, per group, P<0.05. (B and C) Area under the curve (AUC) after a intraperitoneal glucose tolerance test (IPGTT) 30 days following islet transplant, P = 0.6. Note, IPGTT testing was only carried out on normoglycemic mice, excluding transplanted mice that failed to achieve this endpoint, to avoid mortality. (D) Insulin content (ng/ml) from control and GEE-treated islet grafts, P = 0.8.

For the 24-hour studies, seven mice were transplanted with 300 control islets and seven mice were transplanted with 300 GEE treated islets. This islet mass was sufficient enough to allow us to view the graft histologically. It was harvested after 24 h and analysed by TUNEL staining. The graft showed a lower percentage of apoptotic islets in the group which received islets treated with GEE when compared to the group which received untreated islets (Fig. 4, Control 23.3±2.6% vs GEE 8.3±0.8%, P<0.05).

The animals that remained normoglycemic underwent IPGTT (diabetic animals were not tested). Although we observed a slight decrease on the area under the curve (AUC) in the group which received islets treated with GEE, there were no significant differences between the groups (Fig. 4, P = 0.58).

Removal of kidneys bearing islet grafts 30 days after transplantation in the normoglycemic animals produced hyperglycemia, indicating that the islet grafts were responsible for the correction of diabetes. Histological analysis of the 30 day islet grafts showed a low percentage of TUNEL positive cells, with no significant difference between the groups (Control 2.5% vs GEE 2.0%, p  = 0.89). All grafts used in the analysis were obtained from cured mice, five transplanted with 150 control islets and seven transplanted with 150 GEE treated islets.

It was not possible to demonstrate significant differences in the insulin content on grafts (Fig. 4, Control 1,688±449 vs GEE 2,594±741 ng/ml, p = 0.77). These results were obtained from analysis of the remaining grafts (twelve from the control group and nine from the treated group).

### Human Islets

First, the concentration of 10 mM was tested in three different pancreases. The data provided by membrane integrity (Control 70.8±3.6% vs GEE 81.6±6.6% p = 0.027) suggest an improvement in the GEE group after 24 h culture, however, the analyses by TUNEL (Control 46.0±6.8% vs GEE 35.7±4.9%, p = 0.23), fractional beta-cell viability (Control 30.4±6.3% vs GEE 29.3±6.2, p = 0.23), ROS content (Control 65.2±12.2% vs GEE 65.0±11.1%, p = 1.0) and insulin stimulation index (Control 0.7±0.1 vs GEE 1.0±0.1, p = 0.12) did not show significant differences between the groups.

**Figure 5:**
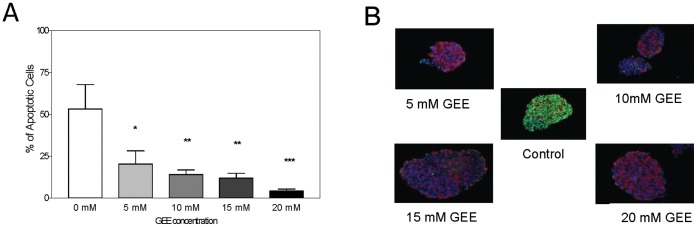
TUNEL Positive Cells. (A) Measurement of apoptotic cells in human islets after culture for 24 hours in the absence (Control) or presence of different GEE concentrations. *p<0.05; **p<0.01; †*p<0.001 in comparison to control* (n = 2 isolations). (B) Microscopic TUNEL staining images of representative islets according to GEE concentration (apoptotic cells = green, insulin staining = red and nucleus = blue).

Looking for an optimal dose to be used in human islet cultures, samples of two pancreases were submitted to different GEE concentrations during 24 h culture. The results observed for membrane integrity, fractional beta-cell viability and ROS content are summarized in Table 1. There were no significant differences among the groups except for the lower rate of TUNEL-positive cells in all tested concentrations of GEE in comparison to untreated islets (Table 1 and Figure 5).

**Table 1:** Human islets after 24 h culture on different GEE concentrations.

GEE concentrations	Control	5 mM	10 mM	15 mM	20 mM
Intracellular ROScontent (%)	91.7±2.8	90.6±2.2	90.5±0.3	88±0.3	87.7±4.9
Membraneintegrity (%)	67.9±7.9	75.9±0.1	91.7±16.8	58.6±8.4	77.6±1.1
Viable beta-cells (%)	38.7±12.9	43±13.3	42.1±6	41.9±3.8	48.5±5
TUNEL-positivecells (%)	53.2±14.5	20.3±7.8*	14±2.7**	12±2.9**	4.3±1.1***

Intracelullar ROS was evaluated using carboxy-H_2_DCFDA and flow cytometry. Membrane integrity was evaluated by Syto green/Ethidium bromide. Fractional beta-cell viability was assessed using Newport Green, 7-AAD and TMRE.

* *p*<0.05;

** *p*<0.01;

*** *p*<0.001 in comparison to control.

## Discussion

To date, the replacement of ß-cells by pancreas and pancreatic islet transplantation are the only concrete alternatives for re-establishing the endogenous insulin secretion in patients with type 1 diabetes. The Edmonton Protocol [1] introduced several modifications to the transplantation procedure which significantly improved 1-year outcomes. Although preliminary results of a 5-year follow-up demonstrated rates of insulin independence that exceed 50% in centers with great experience in islet transplantation [23], further advances in the preservation of the function of isolated islets are still necessary.

While many factors such as the graft implantation site, inflammatory response, allo-rejection and auto-immunity play a role in decreased long-term graft function, studies have shown that losses can be evidenced in the first moments following isolation and transplantation [3], [4]. The isolation process provides a very hostile environment [6], certainly associated with ROS overproduction, cellular redox imbalance and cell death [25]. Pancreatic beta cells appear to be particularly vulnerable to oxidative stress since they have lower antioxidant capacity compared to other cell types [26]. Animal studies have shown that pancreatic islets present lower activity of the main endogenous antioxidant enzymes superoxide dismutase, catalase and GSH peroxidase [26], [27] and Tonooka et al. showed very low concentrations of GSH peroxidase in human islets isolated for transplantation (18).

There is evidence suggesting that the use of GSH improves the oxidative damage associated with ischemic injuries [12], [15], [28]. However GSH is not efficiently transported into most animal cells [20] while GEE is able to raise the intracellular level of GSH [20], [29], [30], because the addition of an ester group to GSH allows the molecule to be delivered directly into cell [31], [32]. Some benefits of GEE have already been demonstrated in animal models of pancreatitis [33] and stroke [15].

To our knowledge, this is the first study to assess the impact of GEE supplementation on the outcomes of islet isolation and transplantation. GEE was able to decrease the content of ROS in isolated islets, reducing the rate of apoptosis while maintaining mitochondria integrity. This integrity, measured by TMRE staining, has a central importance because islet mitochondria plays a crucial role on islet viability and loss of mitochondrial transmembrane potential triggers the intrinsic pathway of apoptosis by activation of effector caspases [34]. The improved beta cell viability observed *in vitro* was translated into better outcomes *in vivo*, since supplementation of GEE during the isolation process resulted in a significantly lower rate of apoptosis in the islet grafts recovered after 24 h of transplantation and in a higher percentage of normoglycemia in mice transplanted with a marginal islet mass in comparison to mice transplanted with untreated islets.

The findings of the present study are in line with others previously published; Armann et al. demonstrated that levels of ROS in islets correlate with the percentage of apoptotic cells and their functional potency *in vivo* [25]; Avila et al. reported that addition of glutamine, a precursor of GSH, during pancreas distension was effective in enhancing islet viability [19], [35]; Lepore et al. described that increased expression of GSH peroxidase protected islet beta cells from hypoxia-reoxygenation damage [36] and Mysore et al. showed that transgenic mice overexpressing GSH peroxidase and superoxide dismutase had a better outcome after marginal mass islet transplant [37].

Supplementation of GEE during the isolation process increased islet viability without significantly modifying glucose-stimulated insulin release. However, Littman et al. have demonstrated that incubation of isolated islets with 10 mM GSH enhanced their secretory response to glucose stimulation [38]. Thus, it is possible that the ideal intervention would be supplementation of GEE not only during islet isolation, but also during their maintenance in culture until the transplantation procedure.

Because human islets are not regularly released for research until several hours after isolation, obtention of recently isolated islets is limiting and the experiments with human islets were restricted to *in vitro* studies of cultured islets, without the addition of GEE supplementation at the time of collagenase pancreatic perfusion. Thus, islets were exposed to GEE several hours after isolation. In the first three pancreas preparations tested with 10 mM GEE, a significant difference was observed only in the membrane integrity assay. In the following two pancreases exposed to different GEE concentrations there was a significant reduction only in the rate of TUNEL positive cells. These observed inconsistencies in the results of the different assays probably reflect the heterogeneity of the human islet samples, such as time in culture before exposition to GEE. We therefore cannot definitively conclude regarding the impact of GEE supplementation upon protection of human islets, although these preliminary data seem promising. Future proposed studies should address the potential protective role of GEE exposure during human pancreatic distension, if the current mouse studies are to be extended in to the clinical setting.

In conclusion, the current data corroborate that ROS production is a relevant cause of cellular damage during islet isolation and that the use of antioxidants to protect islets from oxidative cell injury is a rational approach. The supplementation of GEE might be considered a potential intervention to improve islet viability before islet transplantation.
